# Hematometrocolpos in a Pubescent Girl with Abdominal Pain

**DOI:** 10.5811/cpcem.2017.3.33369

**Published:** 2017-07-06

**Authors:** Haleigh C. Kotter, Daniel Weingrow, Caleb P. Canders

**Affiliations:** UCLA Medical Center, Department of Emergency Medicine, Los Angeles, California

## Abstract

Hematometrocolpos is a rare congenital abnormality of the female urogenital system that leads to an imperforate hymen and subsequent retrograde menstruation. We present the case of a 14-year-old female patient who presented to the emergency department with amenorrhea and abdominal pain, and was found to have an imperforate hymen and hematometrocolpos on trans-abdominal point-of-care ultrasound. It is important for emergency physicians to consider this diagnosis in pubescent female patients presenting with abdominal pain, as missed diagnosis can lead to infertility and other complications.

## INTRODUCTION

Hematometrocolpos is a rare congenital abnormality that is infrequently diagnosed in the emergency department (ED). The hymen, which is part of the urogenital sinus, fails to perforate during genitourinary development in approximately one in 2,000 females.^1^–^2^ Patients subsequently develop retrograde menstruation and hematometrocolpos. The classic presentation of hematometrocolpos is a pubescent female who presents with episodic, cramping lower abdominal and pelvic pain. We present the case of a young female patient who presented with abdominal pain and vomiting, and was found to have hematometrocolpos complicated by hydronephrosis bilaterally on transabdominal point-of-care ultrasound (POCUS). Emergency physicians should consider imperforate hymen with hematometrocolpos in the differential diagnosis of pelvic pain in young female patients, as physical exam and POCUS can lead to rapid diagnosis and treatment and prevent complications of the disease, including infertility.

## CASE REPORT

A 14-year-old female patient presented to the ED with intermittent lower abdominal pain for three weeks. Her pain was bilateral and cramping, with episodes of intense worsening that were associated with non-bloody, non-bilious emesis. She reported abdominal distension, constipation, and breast tenderness, and denied urinary changes, vaginal bleeding, or discharge. She had not yet experienced menarche and she denied sexual activity. The patient had seen her pediatrician at symptom onset, and it was documented that the patient had Tanner Stage IV development but no menstruation. No genitourinary exam had been performed. At that visit, the patient was diagnosed with constipation and counseled on diet changes. Two weeks later, the patient returned to her pediatrician for the same symptoms, and an outpatient transabdominal US was ordered to evaluate her pelvic organs. Her pain worsened prior to her scheduled US, so she presented to the ED.

The patient was afebrile with a pulse of 105 beats per minute, blood pressure of 125/91 mmHg, respiratory rate of 18 breaths per minute, and oxygen saturation of 100%. She was actively vomiting and reporting abdominal pain. Abdominal examination was notable for mild distension, suprapubic tenderness, and a palpable mass in the lower abdomen and pelvis. Bowel sounds were normal. Pelvic examination was notable for a bulging, tense, blue, imperforate hymen. Transabdominal POCUS showed a distended vaginal canal, markedly enlarged uterus, and moderate hydronephrosis bilaterally (Video). Renal function was normal on a chemistry panel. She was diagnosed with hematometrocolpos, and gynecology was consulted. The patient was taken to the operating room for hymenectomy, which drained 900 mL of dark brown fluid. She recovered without complication and had normal menstruation one month later.

## DISCUSSION

While rare, imperforate hymen and hematometrocolpos are important diagnoses to consider in pubescent females who have not yet menstruated and present with abdominal pain. Patients with this congenital abnormality are typically asymptomatic until menarche. Most patients present symptomatic during menstruation, as the retained blood and endometrial tissue accumulates and distends the vaginal canal (hematocolpos), uterus (hematometra), or both (hematometrocolpos). Patients commonly present with abdominal pain, pelvic pain, and vomiting. Less common symptoms include back pain, constipation, urinary retention, or urinary incontinence.^2^–^4^ Physical exam findings of hematometrocolpos include a palpable pelvic mass and a bulging hymen, which is often blue or white in appearance.

Ultrasonography is the preferred imaging modality to evaluate for hematometrocolpos, given that it is rapidly performed at the bedside and does not expose the patient to radiation. Transabdominal POCUS typically reveals a large, hypoechoic mass with smooth walls just posterior to the bladder. Depending on the extent of dilation of the pelvic organs, the uterus may appear normal or be dilated. The blood and endometrial tissue within the uterus appears hypoechoic.^3^,^5^ In the case of hematometrocolpos, the volume of fluid can be measured to assist the surgical team in preparation for operation. POCUS can also be used to assess for complications of hematometrocolpos, including hydronephrosis (due to compressive effects of the fluid collection) and free fluid in the abdomen from uterine perforation.

Treatment of imperforate hymen consists of surgical repair, ideally after the tissues have undergone estrogen stimulation. An elliptical incision is made in the membrane adjacent to the hymenal ring, followed by evacuation of the blood and tissue. Redundant hymenal tissue is also removed. If the diagnosis of hematometrocolpos is missed or delayed, patients may develop retrograde menstruation, endometriosis, pelvic adhesions, fallopian tube damage, and infertility.^3^,^6^

## CONCLUSION

Hematometrocolpos is a rare congenital abnormality of the female urogenital system that leads to an imperforate hymen and subsequent retrograde menstruation. Complications of hematometrocolpos include abdominal and pelvic pain, hydronephrosis from extrinsic compression of the ureters, and infertility. It is therefore important to consider the diagnosis of hematometrocolpos in young female patients presenting with abdominal pain, which can be confirmed by examination and transabdominal POCUS.

CPC-EM Capsule

What do we already know about this clinical entity?

Hematometrocolpos is caused by an imperforate hymen and subsequent retrograde menstruation. It presents in young women and can have damaging consequences.

What makes this presentation of disease reportable?

Imperforate hymen is a congenital abnormality that is rarely seen in the emergency department. In this case, the diagnosis was made with thorough physical examination and point-of-care ultrasound (POCUS).

What is the major learning point?

Consider the diagnosis of hematometrocolpos in pubescent women with abdominal pain who have the appropriate findings on exam and POCUS.

How might this improve emergency medicine practice?

Early suspicion and confirmation of hematometrocolpos can lead to early treatment, preventing unnecessary pain and complications, including infertility.

## Supplementary Information

Video Bedside ultrasound showing a distended uterus measuring 6.0 × 4.4 × 6.8cm and containing hypoechoic blood and endometrial tissue, consistent with hematometrocolpos.

## Figures and Tables

**Image:**
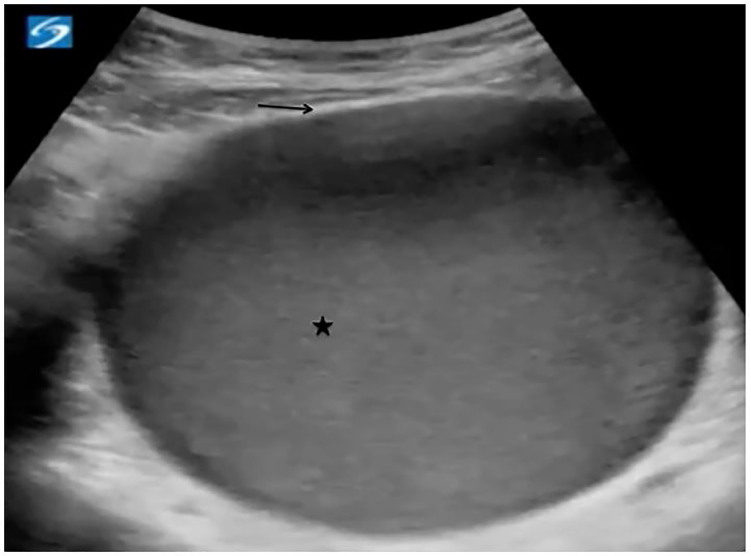
Transabdominal point-of-care ultrasound in transverse view showing a distended uterus measuring 6.0 × 4.4 × 6.8cm and containing hypoechoic blood and endometrial tissue (star), consistent with hematometrocolpos. Internal echotexture of the blood and tissue can be variable, and low-level echoes may be seen. The uterine wall appears hyperechoic (arrow).
